# Association of household income with home-based rehabilitation and home help service utilization among long-term home care service users

**DOI:** 10.1186/s12877-020-01704-7

**Published:** 2020-08-28

**Authors:** Rumiko Tsuchiya-Ito, Tatsuro Ishizaki, Seigo Mitsutake, Shota Hamada, Satoru Yoshie, Katsuya Iijima, Nanako Tamiya

**Affiliations:** 1 Human Care Research Team, Tokyo Metropolitan Institute of Gerontology, 35-2 Sakae-cho, Itabashi-ku, Tokyo, 173-0015 Japan; 2 Dia Foundation for Research on Ageing Societies, Tokyo, Japan; 3 Research Department, Institute for Health Economics and Policy, Association for Health Economics Research and Social Insurance and Welfare, Tokyo, Japan; 4 Health Services Research & Development Center, University of Tsukuba, Ibaraki, Japan; 5 Institute of Gerontology, University of Tokyo, Tokyo, Japan; 6 Department of Health Policy and Management, School of Medicine, Keio University, Tokyo, Japan; 7 Institute for Future Initiatives, The University of Tokyo, Ibaraki, Japan; 8 Department of Health Services Research, Faculty of Medicine, University of Tsukuba, Ibaraki, Japan

**Keywords:** Aging, Economic status, Long-term care services, Claims data, Two-part model

## Abstract

**Background:**

To examine the association of household income with home-based rehabilitation and home help services in terms of service utilization and expenditures.

**Methods:**

A secondary data analysis of cross-sectional design was conducted using long-term care (LTC) insurance claims data, medical claims data, and three types of administrative data. The subjects comprised LTC insurance beneficiaries in Kashiwa city, Japan, who used long-term home care services in the month following care needs certification. Household income was the independent variable of interest, and beneficiaries were categorized into low-income or middle/high-income groups based on their insurance premiums. Using a two-part model, the odds ratios (ORs) and 95% confidence intervals (CIs) for the utilization of home-based rehabilitation and home help services in the month following care needs certification were estimated using logistic regression analysis, and the risk ratios (RRs) of service expenditures were estimated using a generalized linear model for gamma-distributed data with a log-link function.

**Results:**

Among 3770 subjects, 681 (18.1%) used home-based rehabilitation and 1163 (30.8%) used home help services. There were 1419 (37.6%) low-income subjects, who were significantly less likely to use (OR: 0.813; 95%CI: 0.670–0.987) and spend on (RR: 0.910; 95%CI: 0.829–0.999) home-based rehabilitation services than middle/high-income subjects. Conversely, low-income subjects were significantly more likely to use (OR: 1.432; 95%CI: 1.232–1.664) but less likely to spend on (RR: 0.888; 95%CI: 0.799–0.986) home help services than middle/high-income subjects.

**Conclusion:**

Household income was associated with the utilization of long-term home care services. To improve access to these services, the LTC insurance system should examine ways to decrease the financial burden of low-income beneficiaries and encourage service utilization.

## Background

As the global population continues to age, long-term care (LTC) insurance is becoming increasingly important as a social security system for older adults with disabilities [1]. In Japan—the world’s fastest aging country—13.8% of the population was aged ≥75 years in 2017, and this proportion is projected to reach 17.8% in 2025 [2]. Furthermore, the proportion of older Japanese adults receiving LTC services has steadily risen with advancing age [3]. With this rapid increase in the number of LTC insurance beneficiaries, there is a growing pressure to ensure that services are distributed equitably and sustainably according to each individual’s need.

Japan’s LTC insurance system is based on a fee-for-service model, and ostensibly provides freedom of choice regarding services and providers for beneficiaries and their families. However, there are two economic-based limitations to these choices. First, LTC insurance beneficiaries are assigned a care manager, who prepares an LTC plan based on the needs and preferences of each beneficiary and his/her family. Care managers generally select LTC services that do not exceed the allocated limit of service expenditures stipulated for each level of required care and care setting. Second, LTC insurance beneficiaries are charged 10–20% of LTC service costs as out-of-pocket payments (lower-income beneficiaries pay 10%). Therefore, care managers must not only consider each beneficiary’s needs, but also their financial constraints. Due to these limitations, the level of household income is likely to affect LTC service utilization. Although it has been previously reported that unadjusted expenditures for LTC services were higher for low-income beneficiaries than those with higher income [4], another study found that LTC service expenditures for low-income beneficiaries were lower than those with higher income after adjusting for health status–related factors [5]. This difference of expenditures between income level may be due to the regressivity of the out-of-pocket burden, in which out-of-pocket payments account for a higher proportion of household income in lower-income beneficiaries than higher-income beneficiaries [6]. As a consequence, low-income beneficiaries may shy away from using services that are expensive or involve relatively higher minimum payments.

The Japanese LTC insurance system provides both home care services and institutional services, with each service type fulfilling different roles. Home care services include three major service types: home-visit nursing services, home help services, and home-based services provided by rehabilitation professionals (referred to here as “home-based rehabilitation services”) [7]. A previous study reported that LTC beneficiaries with higher income levels were more likely to use home-visit nursing services than those with lower income levels, which may be due to the relatively high costs of these services [8]. As home-based rehabilitation services are also relatively expensive, we hypothesized that low-income beneficiaries may forgo using these services because of insufficient financial resources (Table 1). However, studies have yet to examine the associations of income levels with home help service and home-based rehabilitation service utilization. Home-based rehabilitation services contribute to improved functional capacity [9, 10] and prevent the escalation of care needs [11]. Moreover, LTC insurers in Japan pay an additional fee to home care agencies when home-based rehabilitation and home help service providers work together to enable older adults to perform activities of daily living and lead relatively independent lives [12]. Therefore, this study focuses on both home help services and home-based rehabilitation services.

**Table 1:** Examples of long-term home service unit durations and fees

	Duration of one unit	Fees per unit (USD)^a^
**Home-based rehabilitation services**		
Home-visit rehabilitation from medical facilities or nursing homes	20 min	37.20
Home-visit rehabilitation from visiting nurse agencies	20 min	38.54
Day care services (for persons with care needs level 3)	6–8 h	118.29
**Home help services**		
Physical care	20–30 min	30.98
Daily living support	20–45 min	23.17

^a^Expenditures were converted to USD from JPY (US$1 = ¥82, March 31, 2012)

*LTC* Long-term care.

Furthermore, LTC insurance beneficiaries burdened with high out-of-pocket payments for medical care may forgo the use of LTC services. Accordingly, a beneficiary’s medical expenditures can potentially affect his/her access to LTC services, and it is important to consider these expenditures when examining the association of household income with LTC service utilization. Insurance claims data are frequently used for health utilization and expenditure analyses, but the integrated study of LTC insurance claims and medical claims is hindered by the differing data formats. As a result, few studies have examined the association between household income and LTC service utilization with adjustments for the effects of medical expenditures. In order to contribute to the improvement of access to LTC services in Japan regardless of income, this study aimed to examine if household income was associated with the utilization and expenditures of home-based rehabilitation and home help services.

## Methods

### Study design and setting

This study adopted a cross-sectional design, and was conducted using data from residents of Kashiwa city, Chiba prefecture, Japan. The population, spread over an area of 114.74 km^2^, consisted of 405,099 residents in 2012; of these, 21.3% were aged ≥65 years [13].

### Data sources

We used five data sets: two insurance claims data sets (LTC insurance claims and medical claims) and three administrative data sets (care needs certification for LTC insurance, LTC insurance premium levels, and resident registry data). LTC insurance claims data included the expenditures and amount of LTC services each subject used per month. Medical claims data included each subject’s diagnoses based on International Statistical Classification of Diseases and Related Health Problems 10th Revision (ICD-10) codes, drug prescriptions, and medical expenditures. Data on care needs certification for LTC insurance included each subject’s certified level of required care and his/her degrees of physical and cognitive impairment [14]. In Japan, the levels of required care are categorized into two care support levels (1–2; indicative of the need for preventive care) and five care needs levels (1–5; indicative of the need for LTC), with higher levels signifying greater need. Each beneficiary’s level of required care is determined by computer-based assessment and a panel of specialists appointed by the local government [7]. LTC insurance premium levels were used to indicate household income. Resident registry data included the following socio-demographic characteristics: birth year (provided in five-year brackets to reduce the risk of personal identification), sex, and dwelling type. Anonymous identification numbers were created randomly from insurance numbers for each of the five data sets, and were linked to specific individuals across data sets.

### Subjects

**Fig. 1:**
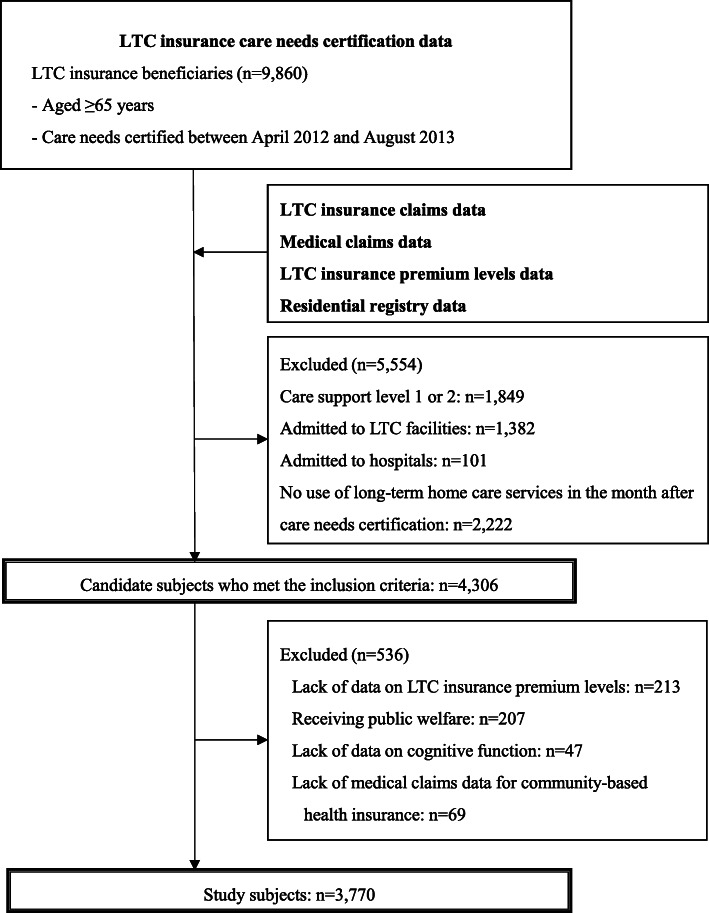
Process of selecting analyzable subjects. LTC = long-term care

The study subjects comprised long-term home care service users aged ≥65 years who were certified as having care needs levels 1–5 by the local government between April 2012 and August 2013. The subject selection process is presented in Fig. 1. From among LTC insurance beneficiaries aged ≥65 years who received care needs certification between April 2012 and August 2013 (*n* = 9860), we excluded those with care support levels 1 or 2 (*n* = 1849), those who were admitted to LTC facilities (*n* = 1382), those who were admitted to hospitals (*n* = 101), and those who did not use any long-term home care services during the month after care needs certification (*n* = 2222). Subjects who did not use any long-term home care services during the month after care needs certification were considered to have no actual or specific need for these services at the time of certification, and were therefore excluded from analysis. After this initial exclusion, there were 4306 candidate subjects.

Next, subjects were excluded if they lacked data on LTC insurance premium levels (*n* = 213), were receiving public welfare (i.e., living below the poverty line as determined by the national government and receiving financial support for all medical and LTC service expenditures under public assistance programs; *n* = 207) [15], lacked data on cognitive function (*n* = 47), or were aged < 75 years with no medical claims data for community-based health insurance (*n* = 69). The final number of study subjects for analysis was 3770.

During the selection process, we were unable to completely exclude all persons aged < 65 years because the birth year data were provided in five-year brackets. Additionally, we only had access to community-based health insurance data; therefore, we excluded people aged < 75 years without community-based health insurance data. For people aged < 75 years, public health insurance in Japan is divided into employee-based and community-based insurance. Employee-based health insurance provides coverage to people (and their dependents) who work at private companies, whereas community-based health insurance provides coverage to people who are self-employed or retired. In contrast, all older persons aged ≥75 years must enroll in the Late Elder’s Health Insurance system [16] and this data was available. Accordingly, all subjects aged ≥75 years were included in analysis.

### Measurements

#### Dependent variables

The dependent variables were the utilization of home-based rehabilitation services and the utilization of home help services in the month following care needs certification. Home-based rehabilitation services comprise home-visit rehabilitation services from medical facilities or nursing homes, home-visit rehabilitation services from visiting nurse agencies, and day care services (Table 1). Home help services comprise physical care, daily living support, and support for getting on and off transit vehicles when visiting a hospital (Table 1); subjects who were provided only one of these services were categorized as using “single-type” services, and subjects who were provided two or more of these services were categorized as using “multiple-type” services.

The utilization of each service was examined based on whether a subject used that service at least once (use or non-use) during the month after care needs certification, as well as the corresponding expenditure. The reason for focusing on service utilization 1 month after certification was that this approach provides insight into each subject’s service needs at the time of care needs certification. Expenditures were calculated by multiplying the frequency of each service used per month by the unit cost (stipulated by the national government). The expenditures included both out-of-pocket payments and the amount paid by LTC insurance. These were converted from JPY to USD using the exchange rate on March 31, 2012 (US$1 = ¥82) [17].

#### Independent variable

The independent variable of interest was household income, which was defined as the total income of each beneficiary and his/her household members. Household income was ascertained from each beneficiary’s LTC insurance premium level, which ranged from level 1 (persons receiving public assistance) to level 16 (persons who are taxed individually with a total annual income ≥10 million yen). Level 4 (persons who are not taxed individually, but have family members paying taxes within the same household) corresponds to the standard household income level where standard premium rates apply. We excluded subjects with level 1 household income as they did not have any available medical claims data. Based on these levels, subjects with levels 2 or 3 (persons and family members who are exempt from resident taxation) were categorized as “low income”, and subjects with levels 4 to 16 were categorized as “middle/high income”. This threshold is frequently used by the Japanese government for policymaking aimed at reducing the economic burden of financially disadvantaged individuals [18].

#### Covariates

The following socio-demographic variables were used as covariates: age group, sex, dwelling type (detached housing or multiple-unit housing), care needs level, physical function, cognitive function, and chronic diseases. Age was grouped into three categories to simulate the age categorization of older people proposed by the Japan Gerontological Society and the Japan Geriatrics Society [19]: 62–71 years (born in 1940 or later), 72–81 years (born between 1930 and 1939), and ≥ 82 years (born in 1929 or earlier). Care needs levels ranged from the lowest (level 1) to the most severe (level 5) [7].

Physical and cognitive function in relation to activities of daily living (ADL) was assessed based on the nationally standardized methods designated by the Japanese Ministry of Health, Labour and Welfare; physical function was assessed using the “degree of independent daily living for older disabled people” and cognitive function was assessed using the “degree of independent daily living for older people with dementia” [14]. Physical function was categorized into “independent” (independent or Level J: patient is able to go out independently), “mild” (Level A: patient requires assistance when going out), “moderate” (Level B: patient requires assistance indoors and sometimes for sitting up), and “severe” (Level C: patient is bedridden). Similarly, cognitive function was categorized into “independent” (independent or Rank I: patient is able to live independently), “mild” (Rank II: patient can generally live independently under observation despite some daily-life–disturbing symptoms, behaviors, and communication problems), “moderate” (Rank III: patient requires assistance in daily life), and “severe” (Rank IV: patient requires frequent assistance and Rank M: patient has marked psychiatric symptoms requiring expert management).

Using ICD-10 codes, we included the following five chronic diseases that represent the main causes of LTC insurance certification: cerebrovascular disease (ICD-10 codes: I60-I63, I69), joint disorders (M15-M17, M19, M43, M47, M48, M50, M51), heart disease (I20–25, I48), dementia (F00-F02, G30, G31), and Parkinson’s disease (G20) [20]. To increase the certainty of the recorded diagnoses, we identified four of these diseases (excluding cerebrovascular disease) using a previously described method based on a combination of ICD-10 codes and the recorded administration of drug classes commonly prescribed to treat these diseases in Japan [21]. We identified individuals with any of the four chronic diseases if their outpatient claims data showed the prescription of a relevant drug class in the same or following month in which the target disease was diagnosed.

The use of home-visit nursing services was also included to adjust for the utilization of each service because home help service users are reportedly more likely to also use home-visit nursing [8]. In addition, beneficiaries may choose to forgo the use of a specific service due to financial constraints, and the utilization of one service may therefore affect the utilization of another. We adjusted for the utilization of home help services when examining the utilization of home-based rehabilitation services, and vice versa.

In patients with financial constraints, the use of medical care may also influence the decision to use LTC services. To account for this potential influence, medical expenditure was included in the analysis. Medical expenditure was defined as the mean medical expenditure for the month of care needs certification and the subsequent month, and was categorized into tertiles: lower group (≤US$142.62), middle group (US$142.63–551.59), and higher group (≥US$551.60).

### Statistical analysis

To examine the association of the socio-demographic characteristics with household income, we determined the number and percentage of subjects for each characteristic; these characteristics were compared between low-income beneficiaries and middle/high-income beneficiaries using the χ^2^ test. Next, to examine the association of the socio-demographic characteristics with home-based rehabilitation and home help service utilization, we determined the number and percentage of subjects for each characteristic, mean and standard deviation of expenditures, median expenditures, and the interquartile range of expenditures; these characteristics were compared between the use and non-use of each service using the χ^2^ test.

We then used a two-part model [22] to estimate the utilization probability and total expenditure of each service. Due to the expectation that there would be many zero values in the expenditures of each service, we selected the two-part model approach because of its usefulness in analyzing zero-inflated outcomes [23]. This allowed the identification and exclusion of subjects who did not use the target service and had no associated expenditures. The first part of the model involved a multivariate logistic regression analysis for all subjects. The effect size was defined for each LTC service as the odds ratio (OR) and 95% confidence interval (95% CI), which indicated the likelihood that low-income subjects would use a service relative to middle/high-income subjects. The second part of the model involved a generalized linear model for gamma-distributed data with a log-link function. The effect size was defined as the risk ratio (RR) and 95% CI, which indicated the likelihood that low-income subjects would spend money on a service relative to middle/high-income subjects. Dementia was excluded as a chronic disease from the two-part model due to the multicollinearity between dementia and cognitive function.

All analyses were conducted using SPSS version 23.0 (SPSS Inc., Chicago, IL), and the significance threshold was set at *P* = 0.05 (two-tailed).

## Results

Table 2 shows the number and proportion of subjects using each service. Of the 3770 study subjects, 18.1% used home-based rehabilitation services and 30.8% used home help services. Among those who used rehabilitation services, 19.2% used home-visit rehabilitation from medical facilities or nursing homes, 11.7% used home-visit rehabilitation from visiting nurse agencies, and 69.0% used day care services. Among those who used home help services, 5.5% used single-type services and 94.5% used multiple-type services.

**Table 2:** Numbers and proportions of subjects using home-based rehabilitation and home help services (*n* = 3770)

	n (%)
**Home-based rehabilitation services**: Use	681 (18.1)
**Type of service**	
Home-visit rehabilitation from medical facilities or nursing homes	131 (19.2)
Home-visit rehabilitation from visiting nurse agencies	80 (11.7)
Day care services	470 (69.0)
**Home help services**: Use	1163 (30.8)
**Type of service**^**a**^	
Single	64 (5.5)
Multiple	1099 (94.5)

^a^ Home help services comprise physical care, daily living support, and support for getting on and off transit vehicles; subjects who were provided only one of these services were categorized as using “single-type” services, and subjects who were provided two or more of these services were categorized as using “multiple-type” services

Table 3 shows the association of socio-demographic characteristics with household income. Approximately 37.6% of subjects were categorized into the low-income group. These subjects were significantly associated with older age, being female, living in multiple-unit housing, care needs levels 1 or 5, and having severe cognitive impairment. Conversely, subjects in the low-income group were less likely to have cerebrovascular disease or heart disease.

**Table 3:** Socio-demographic characteristics by household income (*n* = 3770)

	Total n (%)	Household income		*P* value^a^
		Low income n (%)	Middle/high income n (%)	
**Total**	3770 (100.0)	1419 (37.6)	2351 (62.4)	
**Age (years)**				
62–71	395 (10.5)	125 (8.8)	270 (11.5)	0.003
72–81	1318 (35.0)	474 (33.4)	844 (35.9)	
≥ 82	2057 (54.6)	820 (57.8)	1237 (52.6)	
**Sex**				
Male	1323 (35.1)	273 (19.2)	1050 (44.7)	< 0.001
Female	2447 (64.9)	1146 (80.8)	1301 (55.3)	
**Dwelling**				
Detached house	2952 (78.3)	1056 (74.4)	1896 (80.6)	< 0.001
Multiple-unit housing	818 (21.7)	363 (25.6)	455 (19.4)	
**Care needs level**				
Care needs level 1	944 (25.0)	389 (27.4)	555 (23.6)	0.027
Care needs level 2	1108 (29.4)	393 (27.7)	715 (30.4)	
Care needs level 3	712 (18.9)	257 (18.1)	455 (19.4)	
Care needs level 4	548 (14.5)	194 (13.7)	354 (15.1)	
Care needs level 5	458 (12.1)	186 (13.1)	272 (11.6)	
**Physical function**				
Independent (Independent and Level J)	382 (10.1)	152 (10.7)	230 (9.8)	0.708
Mild (Level A)	1925 (51.1)	718 (50.6)	1207 (51.3)	
Moderate (Level B)	1012 (26.8)	386 (27.2)	626 (26.6)	
Severe (Level C)	451 (12.0)	163 (11.5)	288 (12.3)	
**Cognitive function**				
Independent (Independent and Rank I)	1435 (38.1)	498 (35.1)	937 (39.9)	0.034
Mild (Rank II)	1098 (29.1)	429 (30.2)	669 (28.5)	
Moderate (Rank III)	814 (21.6)	322 (22.7)	492 (20.9)	
Severe (Rank IV and Rank M)	423 (11.2)	170 (12.0)	253 (10.8)	
**Chronic diseases**				
Dementia	1064 (28.2)	402 (28.3)	662 (28.2)	0.910
Cerebrovascular disease	1529 (40.6)	535 (37.7)	994 (42.3)	0.006
Joint disorders	1523 (40.4)	597 (42.1)	926 (39.4)	0.104
Heart disease	1241 (32.9)	429 (30.2)	812 (34.5)	0.006
Parkinson’s disease	247 (6.6)	80 (5.6)	167 (7.1)	0.078
**Home-visit nursing**: Use	372 (9.9)	117 (8.2)	255 (10.8)	0.009
**Medical Expenditures (USD)**^b^				
Lower group (≤$142.62)	1257 (33.3)	502 (35.4)	755 (32.1)	0.060
Middle group ($142.63–551.59)	1257 (33.3)	473 (33.3)	784 (33.3)	
Higher group (≥$551.60)	1256 (33.3)	444 (31.3)	812 (34.5)	

^a^ χ^2^ test

^b^ Expenditures were converted to USD from JPY (US$1 = ¥82, March 31, 2012). Expenditures were calculated as the mean medical expenditure for the month of care needs certification and the subsequent month, and was categorized into tertiles: lower group (≤US$142.62), middle group (US$142.63–551.59), and higher group (≥US$551.60)

As shown in Table 4 and Table 5, the proportion of subjects who used home-based rehabilitation services was significantly higher (*p* < 0.001) in the middle/high-income group (20.2%) than in the low-income group (14.4%). However, the proportion of subjects who used home help services was significantly higher (*p* < 0.001) in the low-income group (35.9%) than in the middle/high-income group (27.8%). The mean ± standard deviation (SD) expenditure for home-based rehabilitation services was 786.2 ± 530.5 USD and the median expenditure was 661 USD. The mean ± SD expenditure for home help services was 1113.9 ± 1145.6 USD and the median expenditure was 623 USD. Both service expenditures had right-skewed distributions.

**Table 4:** Association of socio-demographic characteristics with the utilization of home-based rehabilitation services (*n* = 3770)

	Home-based rehabilitation services					
	Non-use	Use		Expenditures^b, c^		
	n (%)	n (%)	*P* value^a^	Mean ± SD	Median (IQR)	*P* value^d,e^
**Total**	3089 (81.9)	681 (18.1)		786.2 ± 530.5	661 (321–1143)	
**Household income**						
Middle/high income	1875 (79.8)	476 (20.2)	< 0.001	804.1 ± 533.1	681 (324–1169)	0.156
Low income	1214 (85.6)	205 (14.4)		744.4 ± 523.4	642 (313–1056)	
**Age (years)**						
62–71	262 (66.3)	133 (33.7)	< 0.001	835.9 ± 550.3	753 (326–1247)	0.496
72–81	1057 (80.2)	261 (19.8)		764.6 ± 527.4	626 (321–1082)	
≥ 82	1770 (86.0)	287 (14.0)		782.6 ± 524.3	681 (321–1152)	
**Sex**						
Male	1010 (76.3)	313 (23.7)	< 0.001	804.9 ± 532.3	731 (313–1180)	0.441
Female	2079 (85.0)	368 (15.0)		770.2 ± 529.2	641 (322–1111)	
**Dwelling**						
Detached house	2376 (80.5)	576 (19.5)	< 0.001	801.6 ± 545.0	669 (321–1169)	0.207
Multiple-unit housing	713 (87.2)	105 (12.8)		701.0 ± 435.0	596 (321–999)	
**Care needs level**						
Care needs level 1	780 (82.6)	164 (17.4)	0.228	648.6 ± 347.0	601 (331–872)	< 0.001
Care needs level 2	893 (80.6)	215 (19.4)		817.2 ± 389.8	754 (385–1115)	
Care needs level 3	578 (81.2)	134 (18.8)		967.7 ± 593.7	968 (449–1311)	
Care needs level 4	466 (85.0)	82 (15.0)		827.8 ± 650.5	491 (313–1368)	
Care needs level 5	372 (81.2)	86 (18.8)		647.9 ± 591.7	324 (235–883)	
**Physical function**						
Independent (Independent and Level J)	327 (85.6)	55 (14.4)	0.127	695.2 ± 389.2	648 (420–875)	< 0.001
Mild (Level A)	1584 (82.3)	341 (17.7)		826.9 ± 497.5	757 (395–1169)	
Moderate (Level B)	817 (80.7)	195 (19.3)		811.7 ± 589.6	665 (313–1185)	
Severe (Level C)	361 (80.0)	90 (20.0)		631.8 ± 564.1	350 (235–883)	
**Cognitive function**						
Independent (Independent and Rank I)	1074 (74.8)	361 (25.2)	< 0.001	764.2 ± 493.2	642 (321–1120)	0.003
Mild (Rank II)	935 (85.2)	163 (14.8)		852.2 ± 550.9	798 (391–1256)	
Moderate (Rank III)	705 (86.6)	109 (13.4)		854.3 ± 607.2	741 (330–1226)	
Severe (Rank IV and Rank M)	375 (88.7)	48 (11.3)		571.4 ± 488.6	330 (247–648)	
**Chronic diseases**						
*Dementia*						
Present	951 (89.4)	113 (10.6)	< 0.001	779.1 ± 515.7	691 (390–1009)	0.852
Absent	2138 (79.0)	568 (21.0)		787.5 ± 533.8	647 (320–1167)	
*Cerebrovascular disease*						
Present	1182 (77.3)	347 (22.7)	< 0.001	852.5 ± 532.1	754 (374–1244)	< 0.001
Absent	1907 (85.1)	334 (14.9)		717.2 ± 520.7	557 (313–1016)	
*Joint disorders*						
Present	1213 (79.6)	310 (20.4)	0.003	736.4 ± 492.5	635 (313–1102)	0.047
Absent	1876 (83.5)	371 (16.5)		827.7 ± 557.5	705 (332–1181)	
*Heart disease*						
Present	1014 (81.7)	227 (18.3)	0.799	740.6 ± 527.3	626 (313–1044)	0.075
Absent	2075 (82.0)	454 (18.0)		808.9 ± 531.2	709 (324–1169)	
*Parkinson’s disease*						
Present	176 (71.3)	71 (28.7)	< 0.001	784.4 ± 513.0	697 (313–1169)	0.980
Absent	2913 (82.7)	610 (17.3)		786.3 ± 532.9	659 (321–1139)	
**Home-based rehabilitation services**						
Use	–	–	–	–	–	
Non-use	–	–		–	–	
**Home help services**						
Use	966 (83.1)	197 (16.9)	0.231	637.7 ± 482.5	433 (311–959)	< 0.001
Non-use	2123 (81.4)	484 (18.6)		846.5 ± 537.7	756 (364–1218)	
**Home-visit nursing**						
Use	269 (72.3)	103 (27.7)	< 0.001	504.9 ± 425.8	321 (235–642)	< 0.001
Non-use	2820 (83.0)	578 (17.0)		836.2 ± 532.0	753 (370–1176)	
**Medical expenditures (USD)**^b^						
Lower group (≤$142.62)	1015 (80.7)	242 (19.3)	0.178	895.1 ± 549.9	847 (432–1264)	< 0.001
Middle group ($142.63–551.59)	1025 (81.5)	232 (18.5)		774.3 ± 479.0	698 (325–1073)	
Higher group (≥$551.60)	1049 (83.5)	207 (16.5)		672.0 ± 539.2	439 (276–973)	

^a^ χ^2^ test

^b^ Expenditures were converted to USD from JPY (US$1 = ¥82, March 1, 2012). Expenditures were calculated as the mean medical expenditure for the month of care needs certification and the subsequent month, and were categorized into tertiles: lower group (≤US$142.62), middle group (US$142.63–551.59), and higher group (≥US$551.60)

^c^ Expenditures were calculated using only subjects who used each service

^d^ Mann-Whitney *U* test

^e^ Kruskal-Wallis test

*IQR* Interquartile range; *SD* Standard deviation

**Table 5:** Association of socio-demographic characteristics with the utilization of home help services (*n* = 3770)

	Home help services					
	Non-use	Use		Expenditures^b, c^		
	n (%)	n (%)	*P* value^a^	Mean ± SD	Median (IQR)	*P* value^d,e^
**Total**	2607 (69.2)	1163 (30.8)		1113.9 ± 1145.6	623 (269–1605)	
**Household income**						
Middle/high income	1697 (72.2)	654 (27.8)	< 0.001	1134.8 ± 1130.6	668 (281–1669)	0.163
Low income	910 (64.1)	509 (35.9)		1087.1 ± 1165.1	583 (253–1559)	
**Age (years)**						
62–71	285 (72.2)	110 (27.8)	0.173	885.3 ± 989.8	544 (227–1200)	< 0.001
72–81	890 (67.5)	428 (32.5)		956.5 ± 1038.4	527 (245–1292)	
≥ 82	1432 (69.6)	625 (30.4)		1261.9 ± 1219.3	753 (308–2011)	
**Sex**						
Male	949 (71.7)	374 (28.3)	0.012	1024.8 ± 1117.7	547 (233–1459)	0.011
Female	1658 (67.8)	789 (32.2)		1156.1 ± 1156.9	663 (295–1703)	
**Dwelling**						
Detached house	2096 (71.0)	856 (29.0)	< 0.001	1076.9 ± 1122.9	593 (265–1500)	0.144
Multiple-unit housing	511 (62.5)	307 (37.5)		1217.2 ± 1202.3	704 (294–1870)	
**Care needs level**						
Care needs level 1	647 (68.5)	297 (31.5)	< 0.001	504.1 ± 453.4	359 (205–612)	< 0.001
Care needs level 2	800 (72.2)	308 (27.8)		670.0 ± 605.2	477 (212–931)	
Care needs level 3	522 (73.3)	190 (26.7)		1230.3 ± 1039.7	912 (336–2002)	
Care needs level 4	358 (65.3)	190 (34.7)		1732.7 ± 1383.7	1396 (462–3100)	
Care needs level 5	280 (61.1)	178 (38.9)		2114.8 ± 1427.6	1678 (856–3509)	
**Physical function**						
Independent (Independent and Level J)	236 (61.8)	146 (38.2)	< 0.001	439.4 ± 403.6	302 (155–566)	< 0.001
Mild (Level A)	1418 (73.7)	507 (26.3)		823.2 ± 840.2	482 (235–1105)	
Moderate (Level B)	680 (67.2)	332 (32.8)		1388.8 ± 1289.4	855 (340–2199)	
Severe (Level C)	273 (60.5)	178 (39.5)		1982.6 ± 1355.5	1637 (857–3124)	
**Cognitive function**						
Independent (Independent and Rank I)	961 (67.0)	474 (33.0)	0.071	749.4 ± 832.0	459 (226–936)	< 0.001
Mild (Rank II)	760 (69.2)	338 (30.8)		1098.8 ± 1093.2	681 (293–1654)	
Moderate (Rank III)	587 (72.1)	227 (27.9)		1552.7 ± 1327.1	1168 (410–2372)	
Severe (Rank IV and Rank M)	299 (70.7)	124 (29.3)		1745.5 ± 1398.4	1419 (510–2990)	
**Chronic diseases**						
*Dementia*						
Present	780 (73.3)	284 (26.7)	0.001	1400.0 ± 1288.3	877 (336–2171)	< 0.001
Absent	1827 (67.5)	879 (32.5)		1021.5 ± 1080.2	584 (256–2990)	
*Cerebrovascular disease*						
Present	1075 (70.3)	454 (29.7)	0.204	1111.7 ± 1152.3	614 (273–1592)	0.999
Absent	1532 (68.4)	709 (31.6)		1115.3 ± 1142.1	633 (267–1651)	
*Joint disorders*						
Present	1015 (66.6)	508 (33.4)	0.006	1062.1 ± 1137.2	584 (245–1506)	0.040
Absent	1592 (70.9)	655 (29.1)		1154.1 ± 1151.3	671 (308–1678)	
*Heart disease*						
Present	848 (68.3)	393 (31.7)	0.446	1087.0 ± 1063.9	663 (279–1551)	0.661
Absent	1759 (69.6)	770 (30.4)		1127.7 ± 1185.5	604 (265–1673)	
*Parkinson’s disease*						
Present	163 (66.0)	84 (34.0)	0.266	1576.4 ± 1295.5	1218 (517–2510)	< 0.001
Absent	2444 (69.4)	1079 (30.6)		1077.9 ± 1125.8	593 (265–1541)	
**Home-based rehabilitation services**						
Use	484 (71.1)	197 (28.9)	0.231	905.6 ± 905.4	577 (258–1242)	0.079
Non-use	2123 (68.7)	966 (31.3)		1156.4 ± 1184.5	642 (271–1702)	
**Home help services**						
Use	–	–	–	–	–	
Non-use	–	–		–	–	
**Home-visit nursing**						
Use	198 (53.2)	174 (46.8)	< 0.001	1194.2 ± 1153.2	763 (329–1709)	0.105
Non-use	2409 (70.9)	989 (29.1)		1099.8 ± 1144.2	590 (262–1589)	
**Medical expenditures (USD)**^b^						
Lower group (≤$142.62)	906 (72.1)	351 (27.9)	0.001	840.8 ± 856.7	525 (246–1060)	< 0.001
Middle group ($142.63–551.59)	882 (70.2)	375 (29.8)		995.2 ± 1066.8	534 (265–1354)	
Higher group (≥$551.60)	819 (65.2)	437 (34.8)		1435.2 ± 1325.4	893 (310–2352)	

^a^ χ^2^ test

^b^ Expenditures were converted to USD from JPY (US$1 = ¥82, March 1, 2012). Expenditures were calculated as the mean medical expenditure for the month of care needs certification and the subsequent month, and were categorized into tertiles: lower group (≤US$142.62), middle group (US$142.63–551.59), and higher group (≥US$551.60)

^c^ Expenditures were calculated using only subjects who used each service

^d^ Mann-Whitney *U* test

^e^ Kruskal-Wallis test

*IQR* Interquartile range; *SD* Standard deviation

Table 6 shows the association of household income with home-based rehabilitation service utilization and expenditures based on the two-part model. The first part showed that low-income subjects were significantly less likely to use home-based rehabilitation services than middle/high-income subjects (OR: 0.813; 95% CI: 0.670–0.987). In the second part of the model, expenditures for home-based rehabilitation services were found to be significantly lower for low-income subjects than for middle/high-income subjects (RR: 0.910; 95% CI: 0.829–0.999). Moreover, subjects in the middle (OR: 0.782, 95% CI: 0.632–0.968) and higher (OR: 0.590, 95% CI: 0.470–0.741) medical expenditure groups were significantly less likely to use home-based rehabilitation services than those in the lower medical expenditure group. However, medical expenditures were not significantly associated with home-based rehabilitation expenditures.

**Table 6:** Association of household income with home-based rehabilitation service utilization and expenditures

	First part: Home-based rehabilitation service utilization (*n* = 3770)				Second part: Home-based rehabilitation service expenditures (*n* = 681)			
	OR	95% CI		*P* value	RR	95% CI		*P* value
**Household income**								
Middle/high income	1.000				1.000			
Low income	0.813	0.670	0.987	0.036	0.910	0.829	0.999	0.047
**Age (years)**								
62–71	1.000				1.000			
72–81	0.454	0.346	0.596	< 0.001	0.965	0.855	1.088	0.557
≥ 82	0.328	0.249	0.433	< 0.001	1.032	0.910	1.170	0.662
**Sex**								
Male	1.000				1.000			
Female	0.689	0.571	0.832	< 0.001	1.073	0.982	1.171	0.118
**Dwelling**								
Detached house	1.000				1.000			
Multiple-unit housing	0.632	0.500	0.800	< 0.001	0.948	0.846	1.063	0.362
**Care needs level**								
Care needs level 1	1.000				1.000			
Care needs level 2	1.009	0.789	1.292	0.941	1.198	1.065	1.343	0.002
Care needs level 3	1.058	0.783	1.429	0.716	1.537	1.337	1.766	< 0.001
Care needs level 4	0.775	0.533	1.125	0.180	1.385	1.165	1.651	< 0.001
Care needs level 5	1.124	0.700	1.804	0.628	1.416	1.132	1.772	0.002
**Physical function**								
Independent (Independent and Level J)	1.000				1.000			
Mild (Level A)	1.360	0.974	1.897	0.071	1.208	1.030	1.415	0.020
Moderate (Level B)	1.619	1.096	2.391	0.016	1.188	0.988	1.429	0.067
Severe (Level C)	1.982	1.182	3.321	0.009	1.245	0.970	1.598	0.086
**Cognitive function**								
Independent (Independent and Rank I)	1.000				1.000			
Mild (Rank II)	0.557	0.448	0.692	< 0.001	1.084	0.977	1.202	0.128
Moderate (Rank III)	0.476	0.366	0.618	< 0.001	1.100	0.970	1.247	0.137
Severe (Rank IV and Rank M)	0.383	0.264	0.554	< 0.001	0.850	0.711	1.016	0.075
**Chronic diseases**								
*Cerebrovascular disease*								
Absent	1.000				1.000			
Present	1.707	1.427	2.042	< 0.001	1.101	1.011	1.199	0.027
*Joint disorders*								
Absent	1.000				1.000			
Present	1.643	1.358	1.988	< 0.001	0.923	0.842	1.011	0.086
*Heart disease*								
Absent	1.000				1.000			
Present	0.955	0.788	1.156	0.633	0.970	0.886	1.063	0.515
*Parkinson’s disease*								
Absent	1.000				1.000			
Present	2.243	1.641	3.064	< 0.001	0.938	0.815	1.079	0.372
**Long-term care service use**								
*Home help services*								
Non-use	1.000				1.000			
Use	0.877	0.721	1.067	0.189	0.922	0.838	1.014	0.095
*Home-visit nursing*								
Non-use	1.000				1.000			
Use	1.994	1.518	2.621	< 0.001	0.946	0.824	1.085	0.427
**Type of home-based rehabilitation services**								
Home visit rehabilitation from medical facilities or nursing homes					1.000			
Home visit rehabilitation from visiting nurse agencies					0.939	0.799	1.104	0.446
Day care services					2.524	2.172	2.934	< 0.001
**Medical expenditures**								
Lower group	1.000				1.000			
Middle group	0.782	0.632	0.968	0.024	0.970	0.877	1.072	0.548
Higher group	0.590	0.470	0.741	< 0.001	0.922	0.825	1.031	0.154
*C statistic*	*0.714*	*(0.693–0.735)*						

First part: multivariate logistic regression analysis; Second part: generalized linear model for gamma-distributed data with a log-link function

*OR* Odds ratio; *RR* Risk ratio; *95% CI* 95% Confidence interval

Table 7 shows the association of household income with home help service utilization and expenditures based on the two-part model. The first part showed that low-income subjects were significantly more likely to use home help services than middle/high-income subjects (OR: 1.432; 95% CI: 1.232–1.664). However, the second part showed that expenditures for home help services were significantly lower for low-income subjects than for middle/high-income subjects (RR: 0.888; 95% CI: 0.799–0.986). When compared with subjects in the lower medical expenditure group, subjects in the higher medical expenditure group were significantly more likely to use home help services (OR: 1.206, 95% CI: 1.004–1.448) and incur higher expenditures (RR: 1.274, 95% CI: 1.124–1.444).

**Table 7:** Association of household income with home help service utilization and expenditures

	First part: Home help service utilization (*n* = 3770)				Second part: Home help service expenditures (*n* = 1163)			
	OR	95% CI		*P* value	RR	95% CI		*P* value
**Household income**								
Middle/high income	1.000				1.000			
Low income	1.432	1.232	1.664	< 0.001	0.888	0.799	0.986	0.026
**Age (years)**								
62–71	1.000				1.000			
72–81	1.214	0.933	1.581	0.149	1.005	0.838	1.205	0.960
≥ 82	1.080	0.829	1.407	0.567	1.229	1.025	1.475	0.026
**Sex**								
Male	1.000				1.000			
Female	1.112	0.946	1.308	0.199	1.166	1.042	1.306	0.008
**Dwelling**								
Detached house	1.000				1.000			
Multiple-unit housing	1.443	1.220	1.706	< 0.001	1.238	1.107	1.384	< 0.001
**Care needs level**								
Care needs level 1	1.000				1.000			
Care needs level 2	0.930	0.758	1.140	0.483	1.232	1.070	1.419	0.004
Care needs level 3	0.930	0.724	1.194	0.570	2.075	1.743	2.469	< 0.001
Care needs level 4	1.260	0.941	1.688	0.121	2.615	2.132	3.209	< 0.001
Care needs level 5	1.404	0.964	2.044	0.077	3.218	2.504	4.136	< 0.001
**Physical function**								
Independent (Independent and Level J)	1.000				1.000			
Mild (Level A)	0.589	0.460	0.753	< 0.001	1.427	1.216	1.676	< 0.001
Moderate (Level B)	0.690	0.511	0.931	0.015	1.484	1.215	1.812	< 0.001
Severe (Level C)	0.756	0.508	1.126	0.169	1.666	1.284	2.163	< 0.001
**Cognitive function**								
Independent (Independent and Rank I)	1.000				1.000			
Mild (Rank II)	0.857	0.717	1.024	0.089	1.166	1.032	1.316	0.013
Moderate (Rank III)	0.696	0.564	0.859	0.001	1.223	1.057	1.415	0.007
Severe (Rank IV and Rank M)	0.615	0.467	0.810	0.001	1.083	0.896	1.309	0.409
**Chronic diseases**								
*Cerebrovascular disease*								
Absent	1.000				1.000			
Present	0.949	0.818	1.102	0.494	0.955	0.862	1.058	0.375
*Joint disorders*								
Absent	1.000				1.000			
Present	1.170	1.005	1.362	0.043	0.919	0.828	1.020	0.113
*Heart disease*								
Absent	1.000				1.000			
Present	1.024	0.877	1.195	0.763	0.973	0.876	1.081	0.609
*Parkinson’s disease*								
Absent	1.000				1.000			
Present	1.204	0.905	1.603	0.203	1.127	0.929	1.366	0.225
**Long-term care service use**								
*Home-based rehabilitation services*								
Non-use	1.000				1.000			
Use	0.873	0.718	1.061	0.171	0.764	0.667	0.876	< 0.001
*Home-visit nursing*								
Non-use	1.000				1.000			
Use	1.943	1.541	2.450	< 0.001	0.809	0.700	0.934	0.004
**Type of home help service**^**a**^								
Single					1.000			
Multiple					1.621	1.306	2.012	< 0.001
**Medical expenditures**								
Lower group	1.000				1.000			
Middle group	1.014	0.848	1.212	0.877	1.066	0.941	1.208	0.315
Higher group	1.206	1.004	1.448	0.045	1.274	1.124	1.444	< 0.001
*C statistic*	*0.632*	*(0.613–0.651)*						

First part: multivariate logistic regression analysis; Second part: generalized linear model for gamma-distributed data with a log-link function

^a^ Home help services comprise physical care, daily living support, and support for getting on and off transit vehicles; subjects who were provided only one of these services were categorized as using “single-type” services, and subjects who were provided two or more of these services were categorized as using “multiple-type” services

*OR* Odds ratio; *RR* Risk ratio; *95% CI* 95% Confidence interval

## Discussion

This is, to the best of our knowledge, the first study to demonstrate the association of household income with the utilization of home-based rehabilitation and home help services. The study was undertaken to make up for the lack of research on the differences between these two service types. When compared with middle/high-income subjects, low-income subjects were significantly less likely to use or spend money on home-based rehabilitation services. On the other hand, low-income subjects were significantly more likely to use home help services, but less likely to spend money on these services than middle/high-income subjects.

### Household income and home-based rehabilitation services

Low-income subjects were found to have significantly lower utilization and expenditures of home-based rehabilitation services than middle/high-income subjects. This may be because low-income beneficiaries avoid home-based rehabilitation services when choosing LTC services within their allocated expenditure limit. There are two possible reasons for this: first, low-income beneficiaries may have a more present-oriented time preference, i.e., they are more likely to choose immediate utility over delayed utility [24, 25]. Home-based rehabilitation services usually require several weeks or months before meeting the beneficiaries’ minimum expectations, such as improvements in functional capacity. In contrast, home help services can immediately meet the beneficiaries’ needs by supporting their ADL, such as bathing and housework. Thus, even if beneficiaries require both services, those with low household income may not choose home-based rehabilitation services due to their time preference. Another possible reason is the difference in service fees. Our findings were consistent with those of a previous study where LTC insurance beneficiaries in lower-income households used fewer home-visit nursing services than those in higher-income households [8]. Kashiwagi et al. [8] proposed that this was because home-visit nursing is substantially more expensive than home help services. Home-based rehabilitation services are also more expensive than home help services (Table 1), and the higher out-of-pocket payments may discourage low-income beneficiaries from using the former. Moreover, beneficiaries who are near their allocated expenditure limit would be compelled to choose from among these services. In our study’s population, the proportions of beneficiaries that used > 80% of the upper limit of LTC service expenditures were 28.3% in the low-income subjects and 23.4% in the middle/high-income subjects. Accordingly, low-income subjects may eschew home-based rehabilitation services to reduce their out-of-pocket payments.

Our analysis also showed that low-income subjects were less likely to spend money on home-based rehabilitation services. This was consistent with a previous study [5] that reported that lower household income was associated with reduced service utilization. Moreover, the increase in expenditures that accompanied worsening ADL was lower in low-income beneficiaries than higher-income beneficiaries. Sugisawa et al. [5] noted that LTC insurance beneficiaries with lower household income reduced their service-related expenditures to curtail their out-of-pocket spending. The low-income subjects in our study may also have chosen to reduce their use of home-based rehabilitation services to decrease their out-of-pocket burden.

### Household income and home help services

Low-income subjects were significantly more likely to use home help services. Shiba et al. [26] noted that household income is an indicator of social support (e.g., informal caregiving from family members), which low-income beneficiaries tend to lack. We posited that beneficiaries from low-income households would have less family support than those from middle/high-income households. Despite this, low-income beneficiaries may still choose to restrict out-of-pocket payments for home help services, and therefore spend less on such services than middle/high-income beneficiaries. A previous study in the US similarly found that home help service expenditures were lower for older adults with low income than those with higher income [27]. Our findings suggest that low-income subjects may have reduced their home help service utilization to lighten their out-of-pocket payments. Conversely, middle/high-income subjects may have utilized more home help services than their upper expenditure limits allow. The burden of out-of-pocket payments for low-income beneficiaries may influence their choice of home-based rehabilitation and home help services. To lighten this burden and improve access to these services, it may be important to examine ways to decrease the financial burden of low-income beneficiaries.

### Strengths and limitations

The strengths of this study are as follows: First, this study focused on major LTC services in Japan by linking medical and LTC insurance claims data, and included medical expenditures in the analysis. Second, we also examined home-based rehabilitation services, which are provided under the LTC insurance system and constitute a distinctive component of Japan’s LTC services. These services are generally provided as a medical service in Europe [28–30], and are often unavailable in Asian countries [31, 32].

Our findings should be interpreted with consideration to three limitations. First, this study used secondary data without variables that may affect household income and the utilization of home care services. In particular, household income is dependent on the number of household members, and the presence of family caregivers could affect the decision to use home help services. Moreover, we used household income due to its availability, but a beneficiary’s total assets or savings would be more appropriate indicators of his/her economic status [33]. Additionally, we could not take into account the system in which municipalities and medical insurers take over payments for medical and LTC services in cases where a family’s medical and LTC expenditures in the previous year exceed a government-designated limit [34]. This system would help beneficiaries select medical and LTC services according to their needs, but we could not estimate its effects due to the lack of total expenditure data for each’s beneficiary’s household. However, this is still an ancillary system and the number of users would be relatively low. Second, this study used a cross-sectional design, which precludes any inferences on causal relationships. Although LTC service utilization is unlikely to lower a beneficiary’s household income, out-of-pocket payments may affect his/her household income in the low-income group. Finally, the study was conducted in one city (municipality) in a suburban area, and therefore has limited generalizability to other municipalities and LTC insurance beneficiaries aged 65–74 years who do not use community-based health insurance. Nevertheless, these findings could potentially be extrapolated to other municipalities with similar LTC resources and socio-demographic characteristics.

## Conclusion

This study suggests that LTC insurance beneficiaries with low household income may forgo using home-based rehabilitation services. Moreover, they may choose to reduce the amount spent on home-based rehabilitation and home help services to minimize their out-of-pocket payments. Although older adults with low household income only need to pay 10% of expenditures as out-of-pocket payments, these copayments can accumulate to become a considerable burden. Policymakers need to further examine ways to decrease the financial burden of low-income beneficiaries and improve access to LTC services for low-income beneficiaries.

## Data Availability

All relevant data underlying this study are owned by Kashiwa city. There is a contract between Kashiwa city and the Institute of Gerontology, University of Tokyo. The contract stipulates the following; Kashiwa city does not allow the authors to use the data for any purpose other than this study or to provide it to anyone other than the study members without permission from Kashiwa city. Researchers interested in the data used here should contact the author, Dr. Ishizaki.

## Abbreviations

LTC: Long-term care
ADL: Activities of daily living
ICD-10: International statistical classification of diseases and related health problems 10th revision
